# Supplementary material for: Multiplexed PCR to measure in situ growth rates of uropathogenic E. coli during experimental urinary tract infection
Source: bioRxiv. 2025 Apr 28:2024.11.21.624689. Originally published 2024 Nov 21. Preprint. [Version 2] doi: 10.1101/2024.11.21.624689 (PMC11601645; doi:10.1101/2024.11.21.624689)
Supplement: Supplement 1 [file media-1.zip]

Supplementary figures

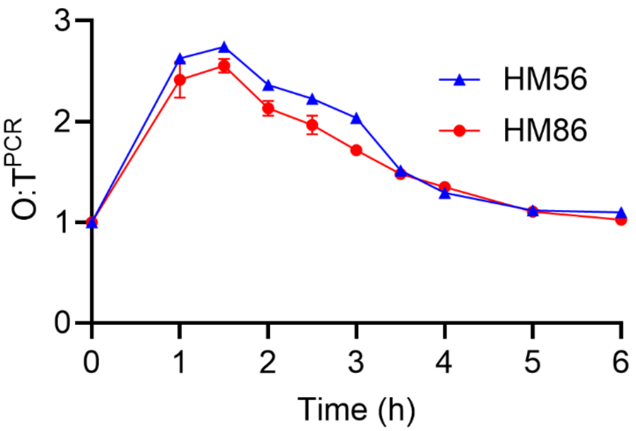

Fig.S1. O:T^{PCR} of the UPEC strains HM86 and HM56 cultured in LB medium. (n=3 for HM86, and n=1 for HM56).

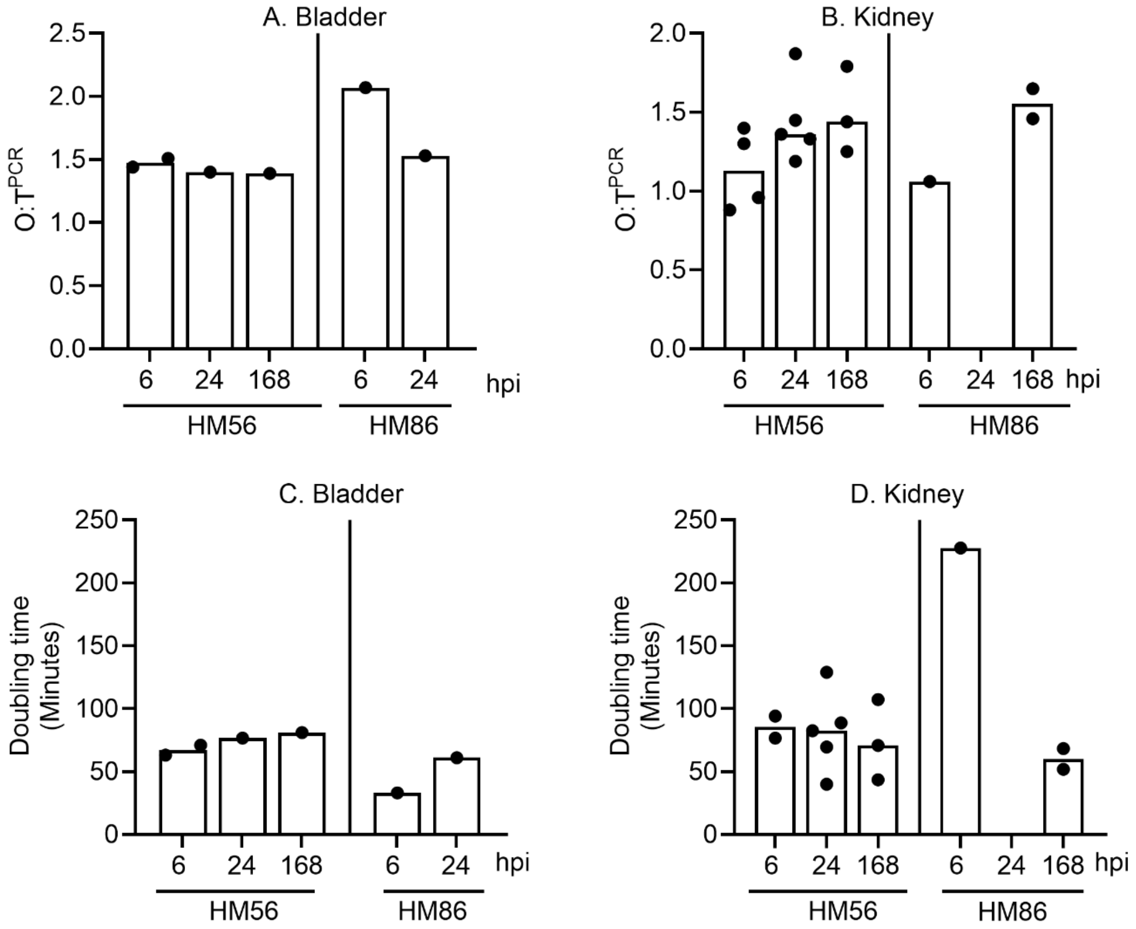

Fig. S2. O:T^{PCR} (Bladder-A, and Kidney-B) and *E. coli* doubling time (Bladder-C, and Kidney-D) of the mouse during experimental UTI at the time point of 6, 24, and 168 hpi.

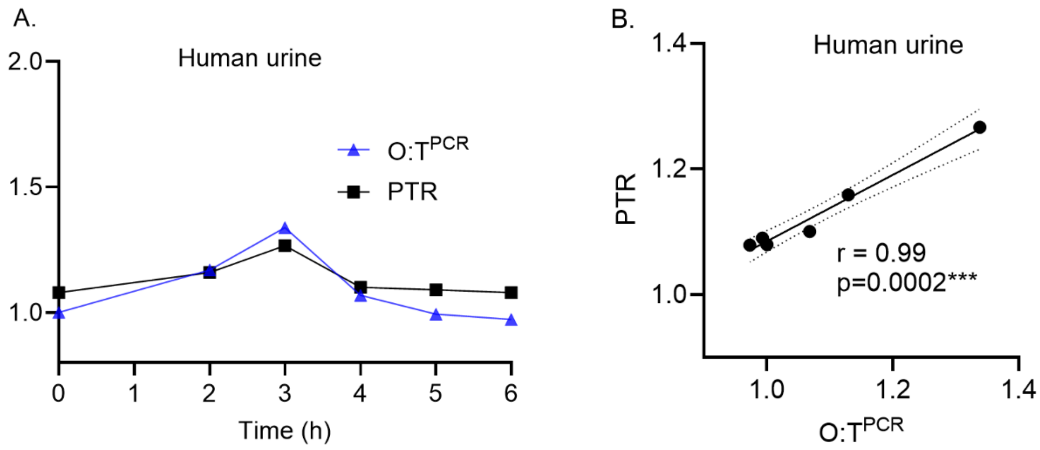

Fig. S3. O:T^{PCR} and PTR of E. coli strain HM86 cultured in human urine (A), and scatterplot with linear regression for the relationship between PTR derived from WGS and *ori:ter* by qPCR method (B). Pearson correlation coefficient (*r*) with *p*-value is shown in the graph. Statistical significance. *** p<.001).
